# Reduction of RUNX1 transcription factor activity by a CBFA2T3-mimicking peptide: application to B cell precursor acute lymphoblastic leukemia

**DOI:** 10.1186/s13045-021-01051-z

**Published:** 2021-03-20

**Authors:** Hélène Jakobczyk, Lydie Debaize, Benoit Soubise, Stéphane Avner, Jérémie Rouger-Gaudichon, Séverine Commet, Yan Jiang, Aurélien A. Sérandour, Anne-Gaëlle Rio, Jason S. Carroll, Christian Wichmann, Michael Lie-a-Ling, Georges Lacaud, Laurent Corcos, Gilles Salbert, Marie-Dominique Galibert, Virginie Gandemer, Marie-Bérengère Troadec

**Affiliations:** 1 Univ Rennes 1, CNRS, IGDR (Institut de génétique et développement de Rennes) - UMR 6290, 35000 Rennes, France; 2 Univ Brest, Inserm, EFS, UMR 1078, GGB, 29200 Brest, France; 3 Département d’onco-hematologie pediatrique, Centre Hospitalier Universitaire de Caen Normandie, Caen, France; 4 CHRU Brest, Service de génétique, laboratoire de génétique chromosomique, 22 avenue Camille Desmoulins, 29238 Brest Cedex 3, France; 5 Department of Hematology, The First Hospital of Jilin University, Changchun, China; 6 Université de Nantes, Ecole Centrale de Nantes, Inserm, CRCINA, Nantes, France; 7 Cancer Research UK Cambridge Institute, University of Cambridge, Cambridge, CB2 0RE UK; 8 Department of Transfusion Medicine, Cell Therapeutics and Haemostasis, Ludwig-Maximilians-University of Munich, Munich, Germany; 9 Cancer Research UK Manchester Institute, University of Manchester, Aderley Park, Macclesfield, SK10 4TG UK; 10 Service de Génétique et Génomique Moléculaire, Centre Hospitalier Universitaire de Rennes (CHU-Rennes), 35033 Rennes, France; 11 Department of Pediatric Hemato-Oncology, Centre Hospitalier Universitaire de Rennes (CHU-Rennes), 35203 Rennes, France

**Keywords:** Childhood leukemia, RUNX1, CBFA2T3, AML1, ETO2, Driver loop, Transcription factor, NHR2, Inhibitor

## Abstract

**Background:**

B Cell Precursor Acute Lymphoblastic Leukemia (BCP-ALL) is the most common pediatric cancer. Identifying key players involved in proliferation of BCP-ALL cells is crucial to propose new therapeutic targets. Runt Related Transcription Factor 1 (RUNX1) and Core-Binding Factor Runt Domain Alpha Subunit 2 Translocated To 3 (CBFA2T3, ETO2, MTG16) are master regulators of hematopoiesis and are implicated in leukemia.

**Methods:**

We worked with BCP-ALL mononuclear bone marrow patients’ cells and BCP-ALL cell lines, and performed Chromatin Immunoprecipitations followed by Sequencing (ChIP-Seq), co-immunoprecipitations (co-IP), proximity ligation assays (PLA), luciferase reporter assays and mouse xenograft models.

**Results:**

We demonstrated that *CBFA2T3* transcript levels correlate with *RUNX1* expression in the pediatric t(12;21) *ETV6-RUNX1* BCP-ALL. By ChIP-Seq in BCP-ALL patients’ cells and cell lines, we found that RUNX1 is recruited on its promoter and on an enhancer of *CBFA2T3* located − 2 kb upstream *CBFA2T3* promoter and that, subsequently, the transcription factor RUNX1 drives both *RUNX1* and *CBFA2T3* expression. We demonstrated that, mechanistically, RUNX1 and CBFA2T3 can be part of the same complex allowing CBFA2T3 to strongly potentiate the activity of the transcription factor RUNX1. Finally, we characterized a CBFA2T3-mimicking peptide that inhibits the interaction between RUNX1 and CBFA2T3, abrogating the activity of this transcription complex and reducing BCP-ALL lymphoblast proliferation.

**Conclusions:**

Altogether, our findings reveal a novel and important activation loop between the transcription regulator CBFA2T3 and the transcription factor RUNX1 that promotes BCP-ALL proliferation, supporting the development of an innovative therapeutic approach based on the NHR2 subdomain of CBFA2T3 protein.

**Supplementary Information:**

The online version contains supplementary material available at 10.1186/s13045-021-01051-z.

## Highlights

- The transcription factor RUNX1 and the transcription regulator CBFA2T3 interact.
- RUNX1 is recruited on its promoter and on an enhancer of *CBFA2T3* located − 2 kb upstream *CBFA2T3* promoter and drives both *CBFA2T3* and *RUNX1* expression.
- CBFA2T3 strongly enhances the transcriptional activity of RUNX1.
- A CBFA2T3-truncated protein functions as a potent inhibitor of RUNX1 and CBFA2T3 protein–protein interaction.
- A CBFA2T3-truncated protein dramatically inhibits RUNX1 transcriptional activity and decreases BCP-ALL lymphoblast proliferation.
- The RUNX1 and CBFA2T3 self-activation loop is a BCP-ALL driver loop.

## Background

RUNX1 (Runt Related Transcription Factor 1) is a major transcription factor of hematopoiesis. It belongs to the RUNX family of transcriptional regulators, where members, RUNX1, RUNX2 and RUNX3, share a *Runt* domain that shows strong evolutionary conservation [1] and is responsible for DNA binding. RUNX1 is essential for definitive hematopoiesis in early development as well as in adulthood for megakaryocyte maturation, T- and B-cell lineage and neuronal development [2–5]. *RUNX1* gene deregulation, either by genetic alterations (point mutation or chromosome abnormalities) or gene expression modification, is involved in many hematological malignancies, notably in ETV6-RUNX1 pre-B acute lymphoblastic leukemia (BCP-ALL) [6–9]. The transcriptional activity of RUNX1 depends on its hetero-dimerization with the non-DNA binding factor CBFβ, and on the recruitment of co-factors [10] that bind functional domains that negatively or positively modulate RUNX1 transcriptional activity [11].

CBFA2T3 (Core-Binding Factor Runt Domain Alpha Subunit 2 Translocated To 3, also known as MTG16, ETO2) belongs to the eight-twenty-one (ETO) family of chromatin-associated proteins. This family also includes Myeloid Translocation Gene 1-Related (MTGR1) and Myeloid Translocation Gene 8 (MTG8, ETO) [12]. Each of these proteins contains four Nervy Homology Region (NHR) domains, and form homo- or hetero-oligomeric ETO complexes via the NHR2 domain [13, 14]. CBFA2T3 is important for hematopoietic stem and progenitor cells self-renewal, lineage commitment and differentiated hematopoietic lineages including T-cell development or erythropoiesis [15–17]. CBFA2T3 participates in oncogenic recurrent translocations in acute myeloid leukemia (with the t(16;21)(q24;q22) giving rise to RUNX1-CBFA2T3) or acute megakaryoblastic leukemia (with inv [16] (p13q24) giving rise to CBFA2T3-GLIS2) [17]. Numerous binding partners have been reported for CBFA2T3, including transcription factors and chromatin modifiers[17], and it is generally believed that the ETO family members act as transcriptional repressor proteins via multiple binding to corepressors, such as nuclear receptor corepressor (NCOR), silencing-mediator for retinoid/thyroid hormone receptor (SMRT), mSin3a, and histone deacetylases (HDACs) [12, 18].

BCP-ALL is the most frequent type of pediatric cancer. Several detailed studies have examined the expression of genes deregulated in ETV6-RUNX1 BCP-ALL compared to other types of leukemia and demonstrated that *RUNX1* and *CBFA2T3* are specifically upregulated in ETV6-RUNX1 BCP-ALL [19] suggesting that these genes could be implicated in the onset and the maintenance of BCP-ALL [19–21]. Moreover, the RUNX1 and CBFA2T3 proteins were previously identified as potential partners in erythroid cells or in an overexpression model in HEK293T cells [22–24].

In this study, we aimed at delineating the functional and structural CBFA2T3 and RUNX1 relationship at the genome and protein levels. First, we demonstrated that RUNX1 protein upregulates *CBFA2T3* gene. Next, we characterized the protein–protein interaction domains between CBFA2T3 and RUNX1. At a mechanistic level, we identified an activation loop between CBFA2T3 and RUNX1. Finally, at a functional level, we demonstrated that a CBFA2T3-mimicking peptide, whose expression was able to disrupt this activation loop by inhibiting RUNX1 activity, results in a decrease of BCP-ALL cell proliferation. Our findings reveal a novel BCP-ALL driver loop dependent on CBFA2T3 and RUNX1.

## Methods

Detailed experimental procedures for RNA extraction, RT-qPCR, generation of stable cell lines, luciferase assay, immunoblotting, chromatin immunoprecipitation (ChIP-Seq), ChIP-PCR and cell cycle and apoptosis assays are presented in Additional file 1. Lists of primers and primary antibodies are presented in Additional file 1: Tables S1 and S2.

### Cell lines and patients’ cells

Pre-B leukemia cell line Nalm6 (ATCC#CRL-3273TM) and REH (ATCC#CRL-8286™) were maintained in RPMI-1640 medium containing 10% heat-inactivated fetal calf serum and 1% penicillin/streptomycin. HEK293 cells (ATCC#CRL-1573TM) were maintained in DMEM/10% fetal calf serum/1% antibiotics. Bone marrow cells from B cell precursor acute lymphoblastic leukemia (BCP-ALL) patients, not presenting the t(12;21) *ETV6-RUNX1* fusion gene, were collected at diagnosis, after informed consent had been obtained, in accordance with the declaration of Helsinki. The protocol was approved by the ethics committee of Rennes Hospital (France).

### Generation of stable cell lines and plasmids

REH^shCBFA2T3^ or REH^shRUNX1^ cells were obtained by transduction with lentivirus MISSION pLKO.1 shRNA puromycine or neomycine resistant (#TRCN0000013660; TRCN0000358353; TRCN0000416005; TRCN0000020165, Sigma-Aldrich). Halotag-ETV6-RUNX1, Halotag-RUNX1 plasmids are described in [25, 26]. CBFA2T3-myc truncated protein plasmids were provided by Andrew Turner and David Callen [27] and subcloned to have Flag-version and to allow lentiviral production in pLL3.7 plasmid for REH^+NHR2^ cell production.

For luciferase assays, genomic DNA fragments derived from the human *CBFA2T3* (chr16:89,045,181–89,045,538) *or RUNX1* (chr21:36,421,428–36,421,673 (hg19 coordinates) gene, and RUNX1-consensus motif repetition AGATTTCCAAACTCTGTGGTTGCCTT (three times repeats) as described in [28] were cloned into *pGL4.10-luc* with a minimal promoter. The *c-KIT* enhancer luciferase plasmid is described in [26].

### Proximity ligation assay (PLA)

PLA was carried out with Duolink® In Situ Detection Reagents (Sigma Aldrich). PLA technology allows the detection of interactions between endogenous proteins, based on the detection of protein proximity. PLA uses one pair of primary antibodies; each antibody targets one of the two distinct proteins for which the proximity is studied. The antibodies used for PLA have been validated by immunofluorescence and western blot. For the validation of each PLA experiment, a negative control (only one type of antibody) and a positive control (two antibodies raised against different epitopes of the same protein are used, this corresponds to ‘total CBFA2T3′ or ‘total RUNX1′ as indicated in the figure legend) are included. The experimental procedure, the automatic quantification and analysis of PLA dots *per* nucleus were extensively described in [25] which is a publication dedicated to the validation of PLA method in pre-B lymphoblasts, especially in non-adherent human pre-B Nalm6 cells, REH cells and human bone marrow mononuclear cells. For the statistical analysis of the results, the cut-off for positivity has been set at two standard deviations over the mean of the negative control signal as described in [25, 29]. To state on the existence or absence of the protein–protein interaction, we performed an analysis of Contingency Table with a Fisher’s exact test comparing the number of dots below and above the cut-off. To state on the variation of the specific number of protein–protein interactions we performed a parametric t-test.

### Chromatin immunoprecipitation (ChIP) and binding site analysis

The detailed procedure is described in Additional file 1. The ChIP-Seq performed are: RUNX1 in REH cells (n = 2), Nalm6 (n = 2), BCP-ALL patients not expressing *ETV6-RUNX1* (n = 3), H3K4me1, H3K4me3, H3K27ac, and CBFA2T3 in REH (n = 1) and Nalm6 (n = 1). Antibodies used are listed in Table S2. All sequencing data are available at NCBI's GEO (https://www.ncbi.nlm.nih.gov/geo/query) through #GSE109377 for RUNX1 in Nalm6 and patients[26] and #GSE117684 for the other data.

### Co-immunoprecipitation (Co-IP) assay

Ten percent (5µL) of protein extracts were separated from the rest for the input. The remaining input was incubated overnight with anti-Flag magnetic beads (M2 clone, Sigma M8823) in a total of 1 mL co-IP Lysis Buffer, on a rotation wheel at 4 °C according to the manufacturer’s recommendations. Ten µL of beads were used for each condition, with a binding capacity of 6 µg of proteins. The following day, the beads were washed with Tris Buffered Saline (TBS) and the proteins were eluted in Laemmli Buffer. Finally, the co-IP samples and their respective input were resolved on a 10% polyacrylamide gel for Western-Blotting.

### Cell proliferation, cell cycle and apoptosis assays

Proliferation assays were done by automatic counting with a Cellometer (Nexcelom). Apoptosis assay was done using Annexin V-FITC (130-092-052, Miltenyi biotec) and Propidium Iodide. For cell cycle analyses, we performed nocodazole block.

### Xenograft transplantation and survival analysis

NOD/*scid* IL2 Rg^*null*^ mice (Charles River Laboratories, France) were maintained in the ARCHE Animal Housing Center (Rennes, France). Animal experiments were performed after authorization by the French Research Ministry, and according to European regulation. Four-week-old mice received two intraperitoneal injections of 20 μg/g busulfan (60 mg/10 mL, Pierre Fabre) on 2 days. They were then allowed to rest for 2 days before retro-orbital injection of 100,000 cells as previously described [30].

### Statistical analysis

Statistical analyses were performed with GraphPad Prism 6 software. Statistical significance was analyzed using Mann–Whitney nonparametric tests for small sizes samples, parametric t-test for larger size samples, and Fisher’s exact test when appropriate.

## Results

### *CBFA2T3* transcript level is upregulated in blasts from BCP-ALL patients and is correlated with *RUNX1* expression.

**Fig. 1:**
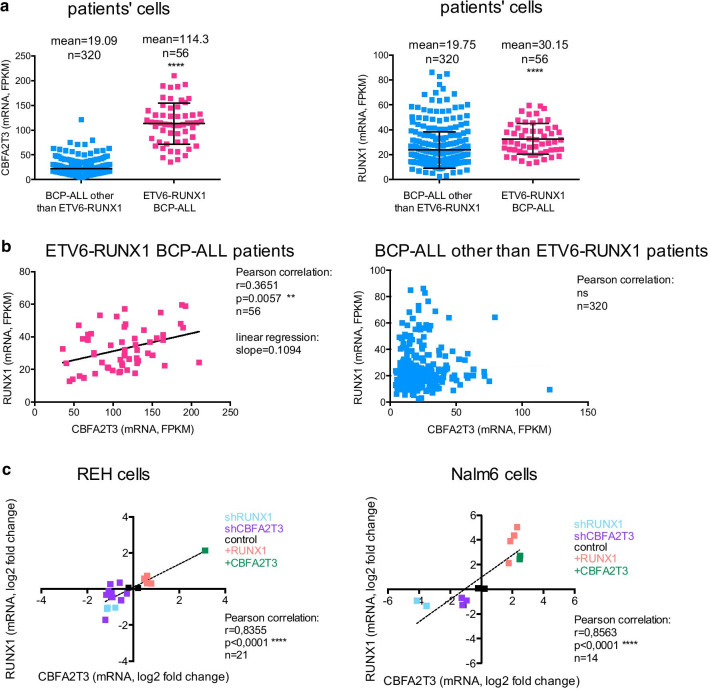
RUNX1 and CBFA2T3 mRNA level are positively correlated in ETV6-RUNX1 BCP-ALL. **a** and **b** The graphs represent the mRNA expression level of *CBFA2T3* and *RUNX1* in human pediatric ETV6-RUNX1 BCP-ALL bone marrow mononuclear cells and the other non-ETV6-RUNX1 BCP-ALL bone marrow mononuclear cells. Data of mRNA levels (expressed in Fragments Per Kilobase Million—FPKM) have been extracted from the St. Jude Children's Research Hospital RNA-Seq Pediatric Cancer Data Portal [31]. For **a** ****p < 0.0001 in t-test between both conditions, for **b** a Pearson correlation has been performed. ns, non significant. Mean data and numbers of samples (n) are indicated above each graph. **c** Correlation between *RUNX1* and *CBFA2T3* mRNA levels originating from REH cells and Nalm6 cells that overexpress or are depleted for RUNX1 or CBFA2T3. Statistical analysis has been performed using Pearson correlation. n, number of samples per condition

Among BCP-ALL subtypes, *RUNX1* and *CBFA2T3* transcripts are upregulated in *ETV6-RUNX1* BCP-ALL blasts (Fig. 1a, Additional file 2: Fig. S1A). This result, obtained from the analysis of the RNA-Seq database of St. Jude Children's Research Hospital Pediatric Cancer Data Portal cohort [31], is coherent with microarray data from previous cohorts [19–21]. Moreover, *RUNX1* transcript level positively and significantly correlates with *CBFA2T3* transcript level in *ETV6-RUNX1* BCP-ALL samples compared to other childhood BCP-ALL (Fig. 1b). Nevertheless, this correlation between *RUNX1* and *CBFA2T3* expression is retrieved under enforced expression or depletion of *RUNX1* or *CBFA2T3* in two BCP-ALL cell lines, the REH cells (that express ETV6-RUNX1 transcript and protein) and Nalm6 cells (that do not express ETV6-RUNX1) (Fig. 1c). First, this result strongly suggests that RUNX1 is involved in *CBFA2T3* expression and vice versa. Second, it also suggests that this correlation is directly dependent on RUNX1 rather than on the presence of ETV6-RUNX1 transcript or protein. We therefore hypothesized the existence of a common activation loop between these two proteins, RUNX1 and CBFA2T3, and further investigated their mutual implication in the control of their transcription and its phenotypic consequence.

### The transcription factor RUNX1 binds *RUNX1* promoter and a *CBFA2T3* enhancer, autoregulates its expression and drives *CBFA2T3* expression

**Fig. 2:**
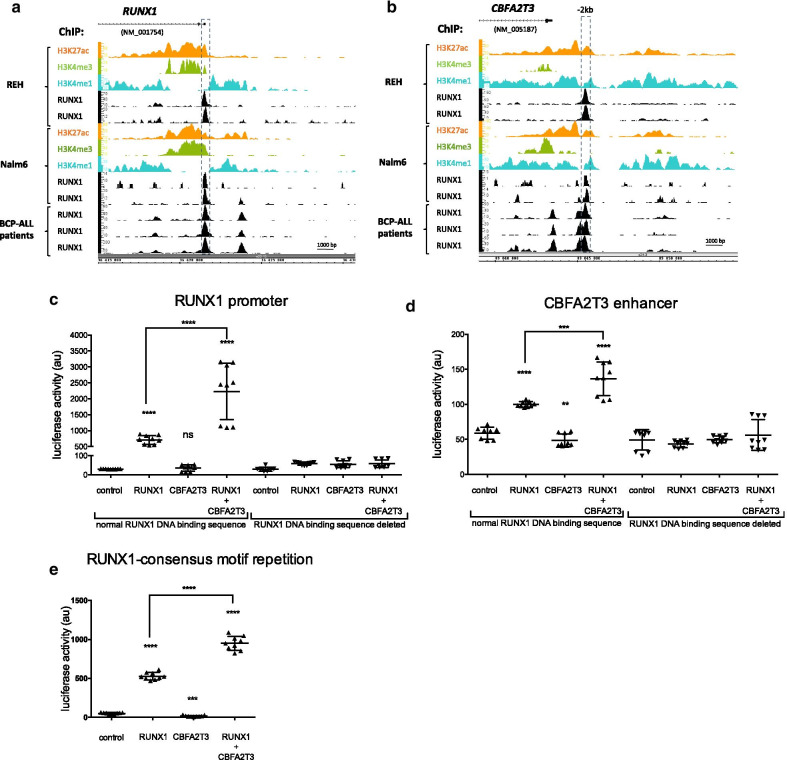
RUNX1 and CBFA2T3 interplay for their transcription. **a** and **b** ChIP-Seq profiles across the human *RUNX1* (**a**) and *CBFA2T3* (**b**) genes. Genomic tracks display ChIP-Seq profiles of RUNX1 from REH cells, Nalm6 cells and BCP-ALL patients, and of the histones H3K27ac, H3K4me3, and H3K4me1 from REH and Nalm6 cells. RUNX1 ChIP-Seq from bone marrow mononuclear cells isolated from three pre-B acute lymphoblastic leukemia patients (BCP-ALL) are also displayed. ChIP-Seq reads were aligned to the reference human genome version GRCh37 (hg19). Each genomic regions of *RUNX1* and *CBFA2T3* genes that are subsequently studied are indicated by boxes. **c**–**e** Luciferase assays with a plasmid containing the *RUNX1* promoter (chr21: 940,581–940,336(C), *CBFA2T3* enhancer (chr16:89,045,181–89,045,538) (D) and a repetition of RUNX1-consensus motif (E) upstream a minimal promoter and a luciferase ORF, in presence of RUNX1 and CBFA2T3 expressing plasmids in HEK293 cells. Note that the HEK293 cells do not endogenously express RUNX1 and CBFA2T3. Luciferase levels (Firefly luciferase/Renilla luciferase) are represented using a scatter dot plot indicating the means and S.D. NS: non-significant, **p < 0.01, ***p < 0.001, ****p < 0.0001 in Mann–Whitney tests compared to the control condition

CBFA2T3 does not bind DNA but acts as a transcriptional regulator. To investigate whether RUNX1 could be recruited to *RUNX1* and *CBFA2T3* regulatory elements (promoters or enhancers)*,* we performed chromatin immunoprecipitation followed by sequencing (ChIP-Seq) with RUNX1 antibody in REH cell line. We compared with our previously published ChIP-Seqs done in Nalm6 cells [26]. We also performed ChIP-Seq with histones H3K4me1 (markers of active enhancers), H3K4me3 (active promoters) and H3K27ac (transcriptionally active chromatin) in REH and Nalm6 cells. RUNX1 occupied 27 786 sites genome-wide in BCP-ALL patients, 5 514 sites in Nalm6 cells with an overlap of 69% between Nalm6 and patients’ cells [26], 9 621 sites in REH cells with an overlap of 60% with Nalm6 and 65% with patients (Additional file 2: Fig. S1B). Among those peaks, we clearly identified that RUNX1 is recruited on its promoter and on an enhancer of *CBFA2T3* located − 2 kb upstream of *CBFA2T3* promoter (Fig. 2a, b). These two bound regions are associated with active chromatin markers suggesting that they are active regulatory elements (Fig. 2a, b).

To confirm the responsiveness of those two RUNX1-bound regions, one found in *RUNX1* promoter and the other in the − 2 kb *CBFA2T3* enhancer, we performed luciferase reporter assays in HEK293 cells overexpressing RUNX1 and/or CBFA2T3 (Fig. 2c, d). The result shows that overexpressed RUNX1 possibly induces transcription from those *RUNX1* and *CBFA2T3* regulatory elements. This transcription is abrogated after deletion of the putative RUNX1 DNA-binding motif found in both regulatory elements, demonstrating the direct implication of the binding of RUNX1 on chromatin on those regions for transcription regulation (Fig. 2c, d).

### The transcriptional regulator CBFA2T3 potentiates the activity of RUNX1

In order to investigate the consequence of CBFA2T3 protein in presence of RUNX1 transcription factor, similarly to what is observed in BCP-ALL patients’ cells (Fig. 1b) and in BCP-ALL cell lines (Fig. 1c), we also tested the impact of CBFA2T3 enforced expression with luciferase assays in HEK293 cells (Fig. 2c, d). Addition of CBFA2T3 does not impact transcription, which is consistent with its inability to bind DNA. However, when both CBFA2T3 and RUNX1 proteins are concomitantly expressed, we observed a synergistic effect on both regulatory elements. This cooperative effect is lost when the putative RUNX1 DNA-binding sequences are deleted (Fig. 2c, d). To definitively ascertain the role of the binding of RUNX1 on its DNA consensus sequence in this cooperation, we used a luciferase assay with a plasmid bearing a tandem of RUNX1-consensus binding sequence as published in [26, 28]. Again, we observed a synergy between RUNX1 and CBFA2T3 for the control of RUNX1-induced transcription (Fig. 2e). Whilst the fusion protein ETV6-RUNX1 overexpression can cooperate with RUNX1 and CBFA2T3 to activate *RUNX1* promoter, it does not drastically impact this cooperation between RUNX1 and CBFA2T3 neither on *CBFA2T3* enhancer nor on the repetition of RUNX1-consensus binding motifs (Additional file 2: Fig. S1c–e), confirming the importance of RUNX1 activity on those regulatory elements over ETV6-RUNX1 activity.

Altogether, our results demonstrated that CBFA2T3 can be an activator of RUNX1 transcription factor. No additional DNA sequence, other than the RUNX1 DNA-consensus sequence, seems to be required to observe the activation of RUNX1-dependent transcription by CBFA2T3. This result orientated us to question the interaction between RUNX1 and CBFA2T3 proteins.

### RUNX1 and CBFA2T3 proteins interact on chromatin

**Fig. 3:**
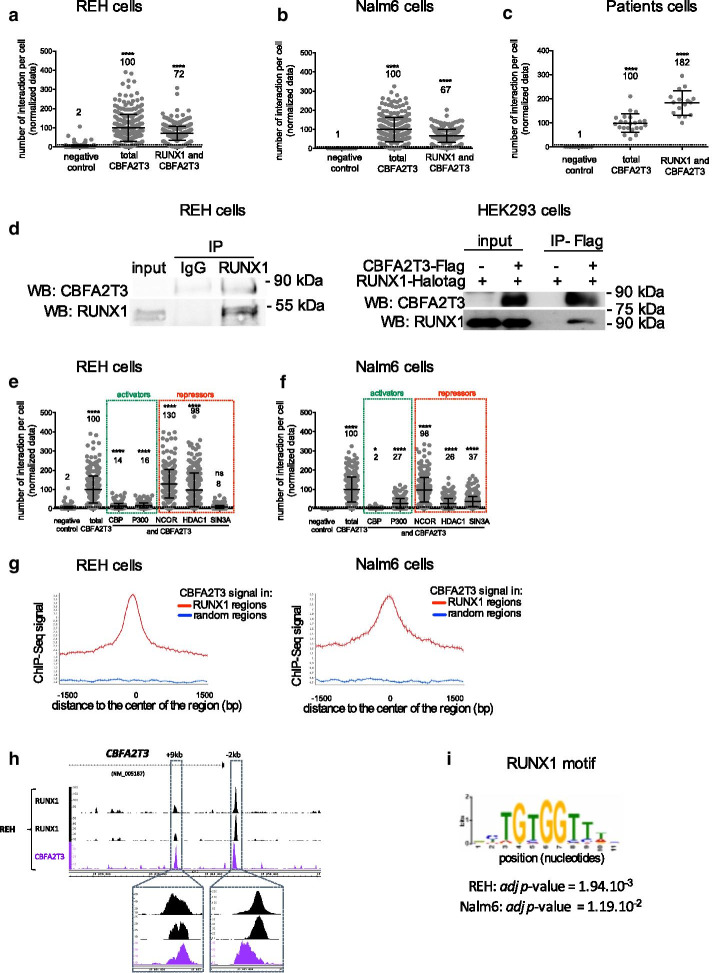
CBFA2T3 and RUNX1 colocalize. **a**, **b**, **c**, **e** and **f** Quantitation of protein co-localization *per* nucleus and visualized by Proximity Ligation Assay (PLA) dots in REH cells (**a**, **e**), Nalm6 cells (**b**, **f**) and BCP-ALL patient cells (**c**), presented with the mean values ± S.D. Antibodies used are indicated under each plot. Positive controls (total CBFA2T3, where primary antibodies against two different epitopes of CBFA2T3 were used) and negative controls (only one anti-CBFA2T3) were included. One representative experiment of at least two independent experiments is shown. The data are normalized against total CBFA2T3. The mean value is indicated above each plot. The positive threshold value is represented by the dotted line (set at two S.D over the background signal as described in [25]). NS: non-significant, * *p* < 0.05, **** *p* < 0.0001 in Fisher’s exact test compared to the negative control condition. **d** Co-immunoprecipitation (co-IP) using (left panel) IgG or RUNX1 antibody in REH cells, and (in right panel) anti-Flag antibody in HEK293 cells expressing RUNX1-Halotag and/or CBFA2T3-Flag plasmids. Western blots were performed with RUNX1 and CBFA2T3 antibodies. Molecular weights are indicated on the right. **g** Density plots of CBFA2T3 Chip-Seq signals into RUNX1-bound regions or random regions in REH and Nalm6 cells. **h** ChIP-Seq profiles across the human *CBFA2T3* gene. Genomic tracks display ChIP-Seq profiles for RUNX1 and CBFA2T3 from REH cells. ChIP-Seq reads were aligned to the reference human genome version GRCh37 (hg19). Two enhancers at + 9 kb and − 2 kb have been focused on. **i** Logo corresponding to the RUNX1 enriched motif for CBFA2T3 regions in REH cells. REH and Nalm6 CBFA2T3 Chip-Seq have been analyzed with the Analysis of Motif Enrichment of the MEME suite [32]. The optimal enrichment *p*-value of the motif according to the Fisher’s exact test, adjusted for multiple tests using a Bonferroni correction is indicated

As CBFA2T3 is neither a transcription factor per se, nor a DNA-binding protein, we suspected that RUNX1 could act as a platform to recruit CBFA2T3 as a co-factor. To look for endogenous protein–protein colocalization, we used a Proximity Ligation Assay (PLA) as we had applied previously to human pre-B cells [25]. RUNX1 and CBFA2T3 endogenous proteins colocalize in the BCP-ALL cell lines REH and Nalm6, and importantly in BCP-ALL patients’ bone marrow blasts (Fig. 3a–c; Additional file 2: Fig. S1F). The interaction between RUNX1 and CBFA2T3 was further confirmed by co-immunoprecipitation in REH cells on endogenous proteins and HEK293 cells with tagged-proteins (Fig. 3D). The interaction is maintained with the fusion protein ETV6-RUNX1 but not with ETV6 demonstrating the role of RUNX1 in this interaction over ETV6 (Additional file 2: Fig. S2a–d). This suggests that RUNX1 and CBFA2T3 could be part of the same regulatory complex. Using PLA, we confirmed the colocalization of CBFA2T3 (Fig. 3e, f) as well as RUNX1 (Additional file 2: Fig. S2E-F) with co-repressors such as HDAC1, NCOR, and SIN3A as already demonstrated by others [13, 18]. Importantly, we also showed that a fraction of RUNX1 and CBFA2T3 colocalize with activators including CREBPP (CBP) and EP300 (P300) (Fig. 3e, f, Additional file 2: Fig. S2E-F). Altogether, these data show that RUNX1 and CBFA2T3 proteins can be part of the same complex, and CBFA2T3 possibly plays an activator role. Whilst CBFA2T3 is not a DNA-binding protein, we performed CBFA2T3 Chip-Seqs in Nalm6 and REH cells. We retrieved 339 CBFA2T3 binding regions in Nalm6 and 2639 in REH cells with 47% (158 peaks) of the peaks in Nalm6 in common with the REH cells (Additional file 2: Fig. S2G). We observed that RUNX1 and CBFA2T3 Chip-Seqs from REH and Nalm6 cells show an enrichment of CBFA2T3 signals in RUNX1-bound regions, as well as an enrichment of RUNX1 signals in CBFA2T3-bound regions, demonstrating the presence of the RUNX1 and CBFA2T3 complex on RUNX1-bound chromatin regions (Fig. 3g, Additional file 2: Figure S2H-I). This is well illustrated in the − 2 kb and + 9 kb enhancers of *CBFA2T3* gene (Fig. 3h), and to a lesser extend on the *RUNX1* promoter by ChIP-qPCR and ChIP-Seq (Additional file 2: Fig. S2J). In total, 5.2% CBFA2T3-bound regions are in common with the RUNX1-bound regions in the REH cells. Concordantly, the RUNX1 consensus binding sequence ‘BYTGTGGTTWB’ is also found significantly enriched, among many others, in the CBFA2T3-bound regions in REH and Nalm6 cells (Fig. 3i, Additional file 3: Tables S3-S4).

Clearly, not all RUNX1-bound regions are shared with CBFA2T3, and not all CBFA2T3-bound regions are shared with RUNX1. This result indicates that RUNX1 and CBFA2T3 are not always in the same complex.

Altogether, our results suggest that a complex between RUNX1 and CBFA2T3 can be poised on chromatin to regulate, and importantly activate, common target genes including *RUNX1* and *CBFA2T3* genes.

### The NHR2 domain of CBFA2T3 is involved in the interaction with RUNX1.

**Fig. 4:**
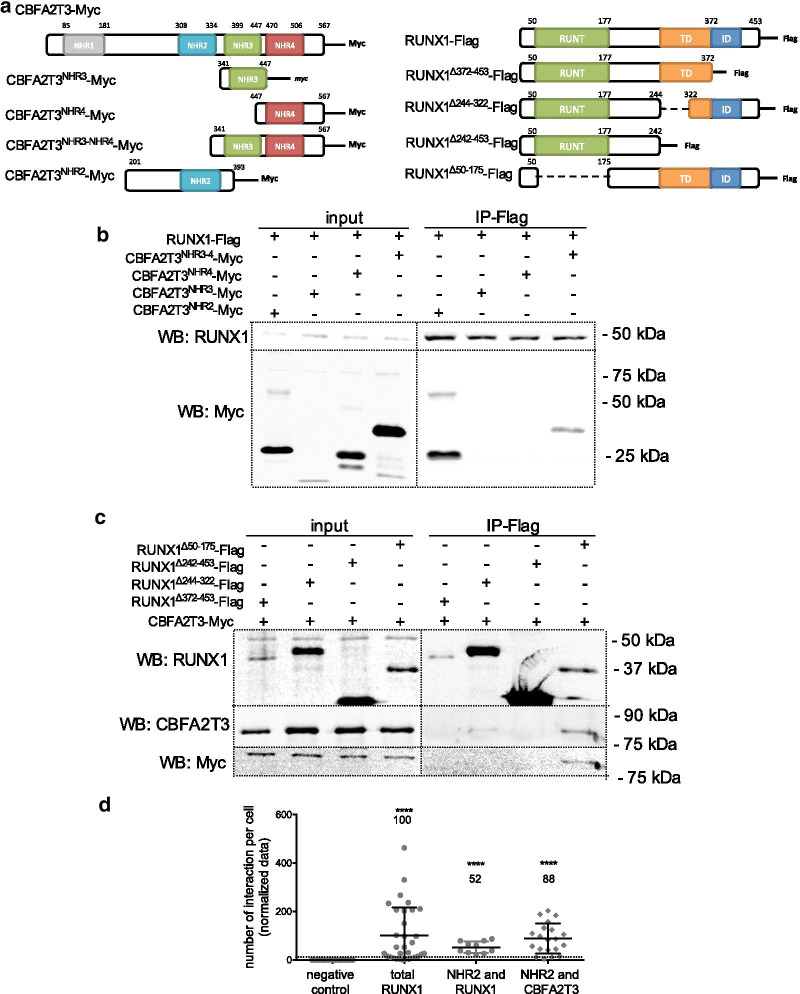
The NHR2 domain of CBFA2T3 is involved in the interaction between RUNX1 and CBFA2T3. **a** Representation of the full-length and truncated CBFA2T3 and RUNX1 proteins used to the functional domain analysis. NHR, Nervy Homology Region; TD, Transactivation domain; ID, inhibitory domain. **b**, **c** Co-immunoprecipitation (IP) using anti-Flag antibody in HEK293 cells expressing various full-length and truncated proteins for CBFA2T3 (**b**) and RUNX1 (**c**) described in (**a**). Western blots were performed with RUNX1, Myc and CBFA2T3 antibodies. Molecular weights are indicated on the right. **d** Quantitation of protein co-localization *per* nucleus and visualized by Proximity Ligation Assay (PLA) dots in HEK293 cells expressing CBFA2T3^NHR2^-Myc with either RUNX1-Halotag or CBFA2T3-Flag, presented with the mean values ± S.D. Antibodies used are anti-RUNX1, anti-Myc and anti-Flag. Positive control (total RUNX1) and negative control were included. The data are normalized against total RUNX1. The mean value is indicated above each plot. The positive threshold value is represented by the dotted line as described in [25]. ****p < 0.0001 in Fisher’s exact test compared to the negative control condition

We further characterized the interaction domain between CBFA2T3 and RUNX1. CBFA2T3 is composed of four functional domains named NHR1 to 4 (Fig. 4a) [27, 33]. RUNX1 displays a highly conserved DNA binding domain (the RUNT domain), followed by a transactivation domain (TD) and an inhibitory domain (ID) (Fig. 4a). Co-immunoprecipations with several CBFA2T3-truncated proteins demonstrated that the CBFA2T3^NHR2^ domain and the CBFA2T3^NHR3−4^ domain interact with RUNX1 in HEK293 cells (Fig. 4b). We also showed that RUNX1^Δ50−175^ and to a much lesser extend RUNX1^Δ244−322^ bind CBFA2T3 suggesting that the C-terminal region (aa372-453) is necessary for binding whereas the RUNT domain and the transactivation domain of RUNX1 are not crucial for this binding (Fig. 4c).

The co-immunoprecipitation of NHR2 and CBFA2T3 was previously reported [34]. Since the CBFA2T3^NHR2^ domain is able to bind RUNX1 and also functions as a tetramerization domain for CBFA2T3, we next confirmed that CBFA2T3^NHR2^ domain not only interacts with CBFA2T3 but also with RUNX1, using proximity ligation assay in HEK293 cells (Fig. 4d). Altogether, our results demonstrated that the NHR2 domain of CBFA2T3 directly interacts with RUNX1.

### Free NHR2 domain of CBFA2T3 functions as a potent inhibitor of endogenous RUNX1 and CBFA2T3 protein interaction

**Fig. 5:**
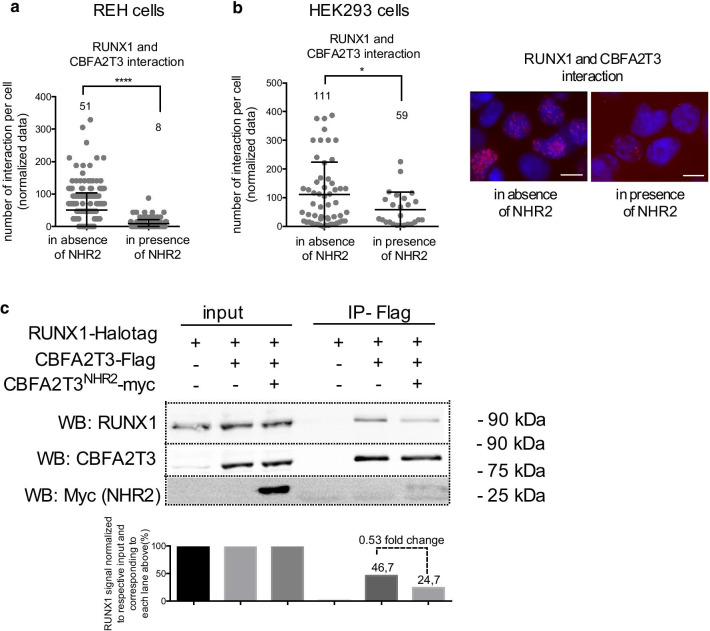
Free NHR2 domain of CBFA2T3 disrupts the protein complex formed by RUNX1 and CBFA2T3. **a** and **b** Quantitation of RUNX1 and CBFA2T3 protein co-localization *per* nucleus and visualized by PLA dots in REH cells (**a**) and HEK293 cells (**b**) in presence or absence of CBFA2T3^NHR2^-Myc, presented with the mean values ± S.D. The mean value is indicated above each plot. The statistical analyses are run with parametric t-tests. *p < 0.1; ****p < 0.0001. The right panel shows a set of pictures of PLA between RUNX1 and CBFA2T3 for HEK293 cells in presence or absence of CBFA2T3^NHR2^-Myc. The PLA dots are in purple, nuclei are in blue (DAPI). Bar: 10 um. **c** Co-immunoprecipitation (IP) using anti-Flag antibody in HEK293 cells expressing RUNX1-Halotag, CBFA2T3-Flag or CBFA2T3^NHR2^-Myc. Western blots were performed with RUNX1, Myc and CBFA2T3 antibodies. Molecular weights are indicated on the right

ETO belongs to the same family as CBFA2T3 and also contains an NHR2 homologous domain. Wichmann et al*.* reported that expression of a 128 amino-acid peptide that includes the NHR2 domain of ETO (NC128) blocks the oligomerization of the RUNX1-ETO fusion protein [35]. Similarly, we hypothesized that ectopic CBFA2T3^NHR2^ domain could act as an inhibitor of the interaction between RUNX1 and CBFA2T3, since we found that CBFA2T3^NHR2^ is able to bind RUNX1 as well as CBFA2T3. Doing so, we observed that the ectopic expression of CBFA2T3^NHR2^ inhibits the endogenous interaction between RUNX1 and CBFA2T3 as assayed by proximity ligation in REH cells (Fig. 5a, Additional file 2: Fig. S2J) and also limits the interaction between exogenous RUNX1 and CBFA2T3 in HEK293 cells (Fig. 5b). Concordantly, by co-immunoprecipitation assay, we demonstrated that the presence of CBFA2T3^NHR2^ decreases by half the interaction between RUNX1 and CBFA2T3 (Fig. 5c). Altogether, our data demonstrated that ectopic NHR2 impedes endogenous RUNX1 and CBFA2T3 protein–protein interaction.

### The NHR2 domain of CBFA2T3 dramatically inhibits RUNX1 transcriptional activity

**Fig. 6:**
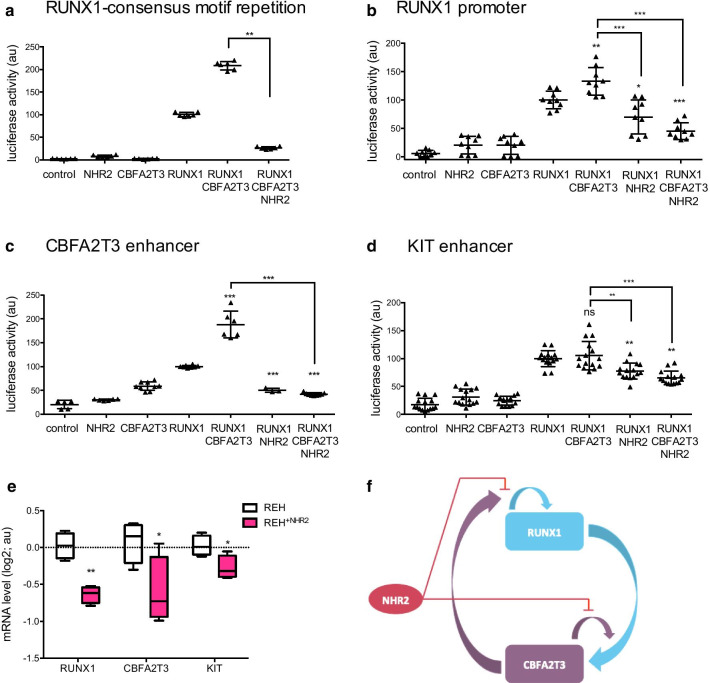
Free NHR2 domain of CBFA2T3 inhibits the cooperative effect of CBFA2T3 on RUNX1 activity. **a**–**d** Luciferase assays with the RUNX1-consensus motif repetition (**a**), the *RUNX1* promoter (**b**), the *CBFA2T3* enhancer (**c**) and the c-*KIT* enhancer (**d**) upstream a minimal promoter and a luciferase ORF, in presence of RUNX1 and CBFA2T3 or CBFA2T3^NHR2^ expressing plasmids in HEK293 cells. Luciferase levels (Firefly luciferase/Renilla luciferase) are represented using a scatter dot plot indicating the means and S.D. ns: non-significant, **p < 0.01, ***p < 0.001, ****p < 0.0001 in Mann–Whitney tests compared to the control condition. **e** Relative mRNA expression of *RUNX1, CBFA2T3* and c-*KIT* measured by RT-qPCR in REH cells, and REH expressing CBFA2T3^NHR2^ cells (REH^+NHR2^). Results are presented in-terms of a fold change after normalizing with *ABL* mRNA. **f** Schematic representation of the activation loop between RUNX1 and CBFA2T3. Expression of the free NHR2 domain interrupts this loop

We next questioned the functional impact of the presence of NHR2 on RUNX1-induced transcription. We first tested the CBFA2T3^NHR2^ truncated protein on the activity of the luciferase plasmid bearing the tandem of RUNX1-binding sequence (Fig. 6a). We observed that the NHR2 totally abrogates the synergistic cooperation between RUNX1 and CBFA2T3 on transcription. Then, we tested whether CBFA2T3^NHR2^ peptide affects transcription of the *RUNX1* promoter and *CBFA2T3* enhancer (used in Fig. 2c, d), and the additional c-*KIT* enhancer, also reported to be RUNX1-sensitive [26], using luciferase reporter assay in HEK293 cells. For these three regulatory elements, addition of CBFA2T3^NHR2^, again, prevents, with various amplitudes, the synergistic activity of RUNX1 and CBFA2T3 on their transcription (Fig. 6b–d). Concordantly with the luciferase assay results, the *RUNX1, CBFA2T3* and c-*KIT* genes targeted by RUNX1 were down-regulated in REH cells expressing CBFA2T3^NHR2^ (Fig. 6e).

Altogether, these results enable us to propose the following model (Fig. 6f). The upregulation of *RUNX1* and *CBFA2T3* could be explained by a cross-activation loop. First, the RUNX1 transcription factor participates in *CBFA2T3* expression. The transcription regulator CBFA2T3, in return, binds RUNX1 protein and enhances *RUNX1* expression, and therefore increases its own expression. We show here that CBFA2T3, often described to have a repressive activity, could also act as co-activator on RUNX1 activity. The presence of intracellular NHR2 blocks this cross-activation loop, by a mechanism that may implicate the competition of NHR2 on RUNX1 and CBFA2T3 bindings.

### NHR2 subdomain decreases lymphoblast proliferation

**Fig. 7:**
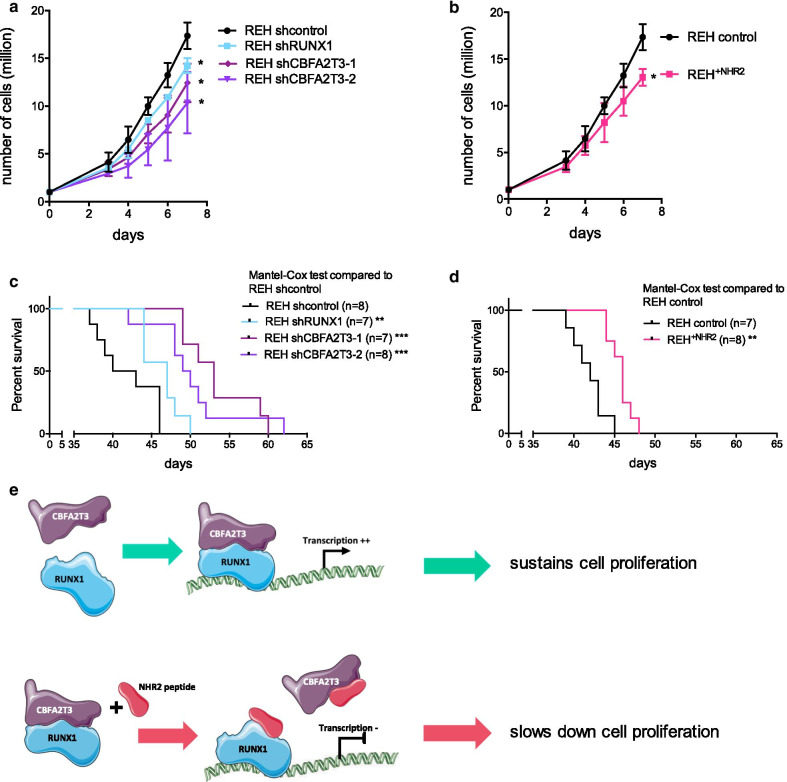
Expression of free NHR2 domain of CBFA2T3 delays BCP-ALL cell proliferation. **a** and **b** Proliferation curves from REH^shcontrol^, REH^shRUNX1^, REH^shCBFA2T3−1^, REH^shCBFA2T3−2^ (**a**), or REH^shcontrol^ and REH^+NHR2^ cells (**b**). Three experiments are represented for each condition. For more readability, statistical analyses have been run only for the last day (day 7) and compared to the condition REH^shcontrol^. * *p* < 0.05. **c**, **d** Kaplan–Meier survival curves from immunodeficient NOD/*scid* IL2 Rg *null* mice xenografted with 100,000 cells of REH, REH^shRUNX1^, REH^shCBFA2T3−1^, REH^shCBFA2T3−2^ (C), or REH^+NHR2^ (D) (n = 7–8 per group). The general condition of mice was monitored daily until experiment ended. Mantel-Cox statistical tests compared to the condition REH^shcontrol^. **p < 0.01, ***p < 0.001. **e** We propose the model in which CBFA2T3 acts as an activator of RUNX1 transcription activity and sustains cell proliferation. Presence of free NHR2 domain disrupts this activation complex and slows down proliferation

We further studied the impact of NHR2 on cell phenotype. Depletions of CBFA2T3 and RUNX1 by stable shRNA decrease cell proliferation suggesting that RUNX1 and CBFA2T3 sustain independently cell proliferation in leukemic cells (Fig. 7a, Additional file 2: Fig. S3A-B). Correlation between mRNA level of *CBFA2T3* or *RUNX1* and the proliferation rate suggests that CBFA2T3 is the main regulator of proliferation in BCP-ALL (Additional file 2: Fig. S3C). Moreover, the combined depletion of CBFA2T3 and RUNX1 (REH^shCBFA2T3+shRUNX1^ cell line, *p* = 0.0010 compared to REH^control^) slows down the proliferation dramatically more than a single depletion of either RUNX1 or CBFA2T3 (REH^shRUNX1+mock^ cell line, *p* = 0.0133 compared to REH^control^ or REH^shCBFA2T3+mock^ cell line, *p* = 0.0280 compared to REH^control^), compatible with additive or cooperative effects between RUNX1 and CBFA2T3 (Additional file 2: Fig. S3D-E). Re-expression of CBFA2T3 is able to rescue half of the proliferation delay induced by the depletion of RUNX1 (REH^shRUNX1+CBFA2T3^ cell line, non significant compared to REH^control^), which is compatible with an additive effect. However, the rescued expression of RUNX1 is not sufficient to restore the proliferation delay induced by depletion of CBFA2T3 (REH^shCBFA2T3+RUNX1^ cell line, *p* = 0.0286 compared to REH^control^), demonstrating that RUNX1 and CBFA2T3 participate into a common pathway, at least partially. Altogether, this demonstrates that CBFA2T3 and RUNX1 bind together to regulate BCP-ALL proliferation, and also points out the importance of the role of CBFA2T3 in BCP-ALL proliferation.

Importantly, expression of NHR2 mimics the depletion of RUNX1 and CBFA2T3 on the reduction of proliferation (Fig. 7b). Moreover, NHR2 reinforces the proliferation delay caused by RUNX1 depletion (Additional file 2: Fig. S3F). The difference in the proliferation rate can be more attributed to moderate differences in cell cycle progression rather than apoptosis that is less than 2% in each of the four cell lines (Additional file 2: Fig. S3G) (data not shown for apoptosis). Mice xenografted with REH cells depleted for RUNX1 or CBFA2T3 (REH^shCBFA2T3^ and REH^shRUNX1^) presented a longer survival compared to mice injected with REH^shcontrol^ cells (Fig. 7c) and similar results are observed with the expression of NHR2 (Fig. 7d). These results demonstrated that NHR2 mimics RUNX1 and CBFA2T3 depletion on proliferative phenotype. The NHR2 domain is an interesting candidate to counteract CBFA2T3 activator effect on RUNX1-induced genes, at the level of the transcriptional activity of RUNX1 as well as at the level of cell proliferation. Our results suggest a model (Fig. 7e) whereby RUNX1 recruits CBFA2T3 to chromatin, enabling a maximal transcriptional activity of the complex. Exogenous presence of NHR2 subdomain prevents the formation of this transcription complex, leading to RUNX1 target genes’ deregulations, and thus slowing down cell proliferation. The NHR2 peptide, or putative drugs mimicking NHR2, could be viewed as an interesting therapeutic tool to slow down BCP-ALL proliferation.

## Discussion

In childhood ETV6-RUNX1 B-cell precursor acute lymphoblastic leukemia (BCP-ALL), the lymphoblasts present altered gene expression. Two genes are overexpressed: *RUNX1* and *CBFA2T3,* and their transcript levels are positively correlated pointing to a common regulator or a common activation loop. Accordingly, RUNX1 and CBFA2T3 proteins were previously identified as potential partners [22–24]. Therefore, we aimed at investigating the physical and functional interactions between RUNX1 and CBFA2T3, and their consequences for leukemogenesis. Our results confirmed that RUNX1 and CBFA2T3 interact, and also showed that RUNX1 binds *RUNX1* promoter and − 2 kb *CBFA2T3* enhancer, and drives the expression of both genes, defining a cross-activating loop. We reported that the regulator CBFA2T3 enhances RUNX1 transcriptional activity. Moreover, we demonstrated that addition of a CBFA2T3-domain NHR2 hampers the interaction between RUNX1 and CBFA2T3, dramatically inhibits RUNX1 transcription activity, and slows down BCP ALL-cell proliferation. Altogether, we demonstrated the existence of a new RUNX1 and CBFA2T3 driver-loop in ETV6-RUNX1 leukemia.

The mechanisms of control of cell specification and differentiation rely on multimeric complexes containing transcription factors, coregulators, and additional non-DNA binding components. The CBFA2T3 transcription regulator is found in such transcriptional complexes in many hematopoietic lineages. At the onset of blood cell specification, the SCL transcription factor forms a multi-protein complex that contains CBFA2T3 and represses cardiac and paraxial cell lineages [36]. In erythroid cells, CBFA2T3 commonly represses GATA-1 function [37], and is critical for the Locus Control Region long-range interactions which support globin genes’ expression [38]]. In myeloid differentiation, CBFA2T3 interacts with PU1 to repress stem cell genes [22]. CBFA2T3 can also partner with PRDM14 on DNA and participates in T-ALL development [39]. Finally, in B-cell lineage, and in Diffuse Large B-cell Lymphoma in particular, CBFA2T3 is reported to be part of the LMO2 complex regulating kinetochore function, chromosome assembly, and mitosis [40]. Our demonstration of the role of CBFA2T3 on the regulation of *RUNX1* expression and function in preB-cells adds to the specificity of this transcription regulator.

In most of those CBFA2T3-multi-protein-containing complexes, CBFA2T3 is described as a repressor. Our data in BCP-ALL cells showed that, indeed, CBFA2T3 is mainly bound to repressor effectors such as NCOR, HDACs and SIN3A. Yet, we also unveiled the binding of a fraction of CBFA2T3 to activators, including CBP and P300. Fujiwara et al*.* reported that the overexpression of CBFA2T3 in erythroid K562 cells leads to the upregulation of 667 genes and the downregulation of 598 genes [41]. It is very likely that among the upregulated genes, there should be genes directly activated by CBFA2T3. The impact of CBFA2T3 on RUNX1 activity, and the consistent observation that CBFA2T3 subdomain inhibitor results in a decrease in expression of *RUNX1, CBFA2T3* and *c-KIT* genes leads us to reconsider the paradigm of CBFA2T3 univocally viewed as a repressor. Here, we show that CBFA2T3 can strongly amplify RUNX1-induced transcriptional activity. The exact mechanism of action, including the precise co-regulators, will be explored in future investigations. Yet, we clearly demonstrated that CBFA2T3 binds RUNX1 transcription factor in an exogenous/overexpression model, but also demonstrated that this binding exists at an endogenous steady-state level in human preB cells, including BCP-ALL cell lines and BCP-ALL primary cells. These results are in agreement with previous reports in erythroid cells [23, 24], or in artificial overexpression model in HEK293T cells [22]. We provide the first demonstration that this RUNX1 and CBFA2T3 complex also occurs in preB-cells, and is responsible for a BCP-ALL driver loop.

We showed, by Chip-Seq, that CBFA2T3 and RUNX1 interaction occurs at the chromatin level, and that CBFA2T3 has a strong impact on RUNX1 transcriptional activity, that is exerted on at least three genes, *RUNX1, CBFA2T3* and *c-KIT*. *CBFA2T3* and *RUNX1* genes are also RUNX1 targets in myeloid cells [42] and in mixed-phenotype acute leukemia [43]. It was also described in mice that Cbfa2t3 itself binds its own promoter in erythroid cell lineage [44], and that RUNX1 binds its promoter in T-ALL cells [45].

We next questioned the role of the interaction between RUNX1 and CBFA2T3 and the self-activation loop between RUNX1 and CBFA2T3 in leukemogenesis. First, it is well known that alteration of each protein, RUNX1 and CBFA2T3, separately, causes hematological malignancies. The fusion protein ETV6-RUNX1 sustains BCP-ALL, RUNX1-ETO is highly frequent in acute myeloid leukemia, RUNX1-MECOM leads to myelodysplasia and acute myeloid leukemia CBFA2T3-GLIS2, and RUNX1-CBFA2T3 is found in acute megakaryoblastic leukemia [6, 7, 17, 46]. These fusion proteins rewire the normal transcriptional program. Globally, mouse models of RUNX1 overexpression do not lead directly to cancer by itself but increases cancer predisposition [47, 48]. CBFA2T3 is required to initiate *Prdm14*-induced T-acute lymphoblastic leukemia, demonstrating its oncogenic role [39]. Even though we demonstrated here an interaction between CBFA2T3 and ETV6-RUNX1 proteins, we did not obtain any evidence that CBFA2T3 could act directly onto ETV6-RUNX1 function. Rather, our data demonstrated the major combined role of CBFA2T3 and RUNX1 proteins in ETV6-RUNX1-containing cells. Our data did not imply either that the presence of ETV6-RUNX1 was required to observe CBFA2T3 and RUNX1 interaction, yet, this interaction is important to sustain cell proliferation of ETV6-RUNX1 cells. Our findings fit perfectly well with the fact that normal RUNX1 is a driver in RUNX1-related leukemogenesis including t(12;21) ETV6-RUNX1 BCP-ALL, t(8;21) AML1-ETO and inv [16] CBFβ-SMMHC acute myeloid leukemia [6, 49]. This role may be exacerbated by the cross-activation loop between RUNX1 and CBFA2T3, where RUNX1 upregulates CBFA2T3, which in turn enhances RUNX1 activity that promotes its own expression. We demonstrated that one mechanism by which CBFA2T3 and RUNX1 promote leukemogenesis or maintain leukemia is by controlling cell proliferation. Our results are concordant with data from Steinauer et al*.* showing that downregulation of CBFA2T3 arrests G1/S cell cycle progression and attenuates in vitro and in vivo proliferation of acute myeloid leukemia cells [50].

We can also question why ETV6-RUNX1 fusion protein could lead to positive correlation of RUNX1 and CBFA2T3 expression. ETV6-RUNX1 results from the fusion of a repressor (ETV6) with almost the entire RUNX1 which is an activator. It has never been formally demonstrated that ETV6-RUNX1 exclusively plays a repressive role on every promoter normally controlled by RUNX1, especially in preB-cells. PLA data in REH cells done with ETV6 antibody (thus targeting ETV6-RUNX1 protein) show that ETV6-RUNX1 colocalizes with activators (CBP, P300) as well as repressors (NCOR, HDAC1) (personal data). At a functional level, in our hands, ETV6-RUNX1 reinforces the activation role of RUNX1 on *RUNX1* promoter (Figure S1C), but not on the *CBFA2T3* enhancer (Figure S1D) or on the RUNX1-consensus motif repetition (Figure S1E) in luciferase promoter assays. This suggests that ETV6-RUNX1 can potentially play a similar activation role than RUNX1, depending on the chromatin context.

We showed that the interaction between CBFA2T3 and RUNX1 mainly occurs by the NHR2 domain of CBFA2T3, which is also its oligomerization domain. The NHR2 domain is a protein–protein interaction motif, and is responsible for homo-oligomerization and dimerization among ETO family members [12, 13]. Ectopic intracellular expression of the NHR2 domain interferes with the RUNX1 and CBFA2T3 complex and decreases proliferation.

Several authors have been able to prevent ETO-fusion or CBFA2T3-fusion protein oligomerization, and importantly have demonstrated a reversion of the leukemic phenotype using a NHR2-mimicking protein or small molecule inhibitors mimicking the tetramerization interface of NHR2, in particular with RUNX1-ETO fusion [14, 51–54] or CBFA2T3-GLIS2 fusion [55]. Our results are in line with these previous works, since we demonstrated that exogenously expressed NHR2 domain prevents RUNX1 and CBFA2T3 interaction, downregulates RUNX1 target genes’ expression and decreases BCP-ALL cell proliferation. However, we cannot exclusively attribute this phenotype to disruption of RUNX1 and CBFA2T3 interactions. Indeed, CBFA2T3 interacts with other partners that can also participate in cell proliferation. Numerous binding partners have been reported for CBFA2T3, including transcription factors (DNA-binding partners) and chromatin modifiers (non-DNA-binding partners) [17]. Few data are available on CBFA2T3 partners in B-cells, but the literature is richer for other hematopoietic lineages. CBFA2T3 depletion arrests cell cycle progression in acute myeloid leukemia cell lines and CD34 + hematopoietic stem and progenitor cells (HSPC) [50, 56]. E-box proteins (E2A/HEB), TAL-1 complexes or GATA1 can be proposed to participate in this proliferation arrest [57, 58], in particular in the erythroid lineage [16, 59].

## Conclusions

Altogether, we described an important activation loop between the transcription factor RUNX1 and the transcription regulator CBFA2T3. We deciphered the interaction of RUNX1 with CBFA2T3, and demonstrated that addition of free CBFA2T3^NHR2^ protein domain inhibits the interaction between RUNX1 and CBFA2T3, and results in reversion of leukemic phenotype by decreasing BCP-ALL cell proliferation. Our findings unveil the role of CBFA2T3 also as a transcriptional activator for RUNX1 in human PreB lymphoblasts, and demonstrate the existence of a new RUNX1 and CBFA2T3 driver-loop in ETV6-RUNX1 BCP-ALL leukemia. Our findings open novel potential therapeutic approach for BCP-ALL.

## Supplementary Information

**Additional file 1: Table S1**. List of primers used in qPCR or ChIP-qPCR. **Table S2: **List of all antibodies used.

**Additional file 2: Figures S1, S2 ** and **S3** and their legends.

**Additional file 3: Table S3**. Analysis of Motif Enrichment for CBFA2T3 regions in REH cells. **Table S4**. Analysis of Motif Enrichment for CBFA2T3 regions in Nalm6 cells.

## Data Availability

The data and materials supporting the conclusion of this study have been included within the article and the supplemental data. The Chip-Seq datasets generated and/or analysed during the current study are available in the NCBI's GEO (https://www.ncbi.nlm.nih.gov/geo/query) through #GSE109377 for RUNX1 in Nalm6 and patients [26] and #GSE117684 for the other data.

## Abbreviations

BCP-ALL: B-Cell Precursor Acute Lymphoblastic Leukemia
CBFA2T3: Core-Binding Factor Runt Domain Alpha Subunit 2 Translocated To 3 (also know as ETO2 and MTG16)
ChIP-Seq: Chromatin Immunoprecipitation followed by Sequencing
Co-IP: Co-immunoprecipitation
NHR: Nervy Homology Region
PBS: Phosphate Buffered Saline
PLA: Proximity Ligation Assay
RUNX1: Runt Related Transcription Factor 1 (also known as AML1)
SDS: Sodium Dodecyl Sulfate
TBS: Tris Buffered Saline
