# Supplementary material for: Reduction of RUNX1 transcription factor activity by a CBFA2T3-mimicking peptide: application to B cell precursor acute lymphoblastic leukemia
Source: J Hematol Oncol. 2021 Mar 20;14:47. doi: 10.1186/s13045-021-01051-z (PMC7981807; doi:10.1186/s13045-021-01051-z)
Supplement: Supplementary file 1 — Additional file 1: Table S1. List of primers used in qPCR or ChIP-qPCR. Table S2: List of all antibodies used. [file 13045_2021_1051_MOESM1_ESM.docx]

**SUPPLEMENTAL MATERIALS AND METHODS**

**Generation of stable cell lines and plasmids**

REH^shCBFA2T3^ or REH^shRUNX1^ cells were obtained by transduction of REH cells in the presence of 8 μg/mL of polybrene (Merck Millipore) with lentivirus bearing MISSION pLKO.1 shRNA-puro or shRNA-neo vectors targeting human RUNX1 (#TRCN0000013660 or TRCN0000358353 Sigma-Aldrich) or CBFA2T3 (#TRCN0000416005 or TRCN0000020165 Sigma-Aldrich, referred respectively in the manuscript as shCBFA2T3-1 and -2). Halotag-ETV6-RUNX1 ORF were subcloned from plasmid kindly provided by G. Nucifora(1). CBFA2T3-Flag human ORF from *pCDNA3-CBFA2T3-Flag,* and Halotag-RUNX1 human ORF from *pFN21A* (#FHC01784, Kazusa collection, Promega) respectively were cloned into a lentivirus *pLenti-CMV-Puro-DEST* by Gateway technology. pLenti-CMV- Puro-DEST (w118-1) was a gift from Eric Campeau (Addgene plasmid #17452)**.** To produce lentivirus, HEK293 cells were co-transfected with *pLenti-CMV-Puro-DEST* bearing plasmids, *pSPAX2* and *pCMV-VSV-G* for packaging using Lipofectamin 3000 transfection reagent (Thermo Fisher Scientific). The plasmid *psPAX2* was a gift from Didier Trono (Addgene plasmid # 12260) and *pCMV-VSV-G* was a gift from Bob Weinberg (Addgene plasmid # 8454). After 48h, supernatant was harvested, filtered and added to REH cells with 8μg/mL polybrene, and transduced cells were selected in medium containing 0.5 μg/mL puromycin (Invivogen). CBFA2T3-myc truncated protein plasmids were provided by Andrew Turner and David Callen(2) and subcloned to have Flag-version. pCMV-CBFA2T3^NHR2^ was subcloned in pLL3.7 vector for REH^+NHR2^ cell production. RUNX1 truncated protein plasmids were provided by Michael Lie-A-Ling and George Lacaud.

**Luciferase assay**

For luciferase assays, genomic DNA fragments derived from the human *CBFA2T3* (chr16:89,045,181-89,045,538) *or RUNX1* (chr21:36,421,428-36,421,673) gene, and RUNX1-consensus motif repetition AGATTTCCAAACTCTGTGGTTGCCTT (three times repeats) as described in (1) were cloned into *pGL4.10-luc* with a minimal promoter and transfected in HEK293T cells, in presence of *pFN-Halotag-RUNX1* and/or *pCDNA3-CBFA2T3-Flag* and supplemented with empty *pCDNA* vector. The luciferase plasmid containing the *c-KIT* enhancer is described in (3). Biological replicates of HEK293T cells were plated into 12-well plates and co-transfected with 0.25 μg of pGL4.10*-luc* plasmid DNA, together with 0.25 μg of the appropriate vector using lipofectamine 3000 (L3000015, Invitrogen). *pCMV-renilla* luciferase vector was also transfected as an internal control for transfection efficiency. Forty-eight hours after transfection, cells were lysed and assayed for luciferase activity using the dual luciferase reporter system (Promega) according to the manufacturer's protocol and a LB 960 Centro luminometer (Berthold technologies).

**RNA extraction, cDNA synthesis, and RT-qPCR**

RNA was extracted using the NucleoSpin RNA II (Macherey Nagel). cDNA was synthesized using High capacity cDNA RT kit (Life Technologies) according to the manufacturer's protocol. Real-time PCR was carried out in sealed 384-well microtiter plates using the SYBR™ Green PCR Master Mix (Applied Biosystems), according to Applied Biosystems gene amplification specifications (40 cycles of 15 sec at 95°C and 1 min at 60°C). The forward (F) and reverse (R) primers (synthesized by Eurogentec) were described in **Table S1.** Data analysis was performed using the ΔΔCT-method (18). The housekeeping gene ABL was used to normalize the data.

**Chromatin immunoprecipitation sequencing (ChIP-Seq) and binding site analysis**

The procedure was previously described(3). Approximately 1.10^8^of REH cells or blasts from BCP-ALL patient were fixed in 1% formaldehyde (648336, Polysciences) for ChIP sequencing) at room temperature for 10 min and then quenched in 100 mM glycine for 1 min. Cells were washed twice in PBS and lysed 15 min in 50 mMTris-HCl pH 8.1/10 mM EDTA/0.5% Empigen BB/1% SDS. Lysates were sonicated to shear DNA to lengths between 200 and 600 base pairs and spun at 10,000 g for 10 min at 4°C. The sonicated cell supernatants were diluted 6-fold in ChIP Dilution Buffer (20 mMtris-HCl pH 8.1/150 mMNaCl/0.1% triton) and incubated with 10 μg for ChIP sequencing for overnight at 4°C (Table S2). Then, 100 μl of magnetic bead protein G (10004D, Invitrogen) and yeast tRNA (R5636, Sigma Aldrich) were added to the lysate for 4 h at 4°C under agitation. After four washings, immunoprecipitated DNA was eluted with elution buffer containing 1% SDS and 0.1 M NaHCO_3_. The protein–DNA crosslinks were reversed by heating at 65°C overnight, and chromatin was cleaned up using PCR purification kit (Qiagen). For sequencing, DNA quality was assessed with the Agilent Bioanalyzer with a High Sensitivity Chip. ChIP-Seq libraries were generated using TruSeq® ChIP Library Preparation Kit (Illumina) according to the manufacturer’s protocol. High-throughput sequencing was conducted on a Genome Analyzer II (Illumina, San Diego, CA) at the Human and Environmental Genomics' platform of Rennes (Biogenouest génomique, Rennes, France). ChIP-Seq reads were aligned to the reference human genome version GRCh37 (hg19) and peak calling were carried out. We performed ChIP-Seq of histones H3K4me1, H3K4me3 and H3K27ac in REH cells and Nalm6. We performed RUNX1 ChIP-Seq: 2 biological replicates for REH cells, 2 biological replicates for Nalm6 cells, 3 with bone marrow leukemia cells isolated from three BCP-ALL patients. CBFA2T3 ChIP-Seq was also performed in REH and Nalm6 Cells. We run the Analysis of Motif Enrichment of the MEME suite version 5.1.1(5).

ChIP-Seq data were acquired by Illumina sequencing and visualized with Integrated Genome Browser 9.0.0(6). All sequencing data are available at NCBI's Gene Expression Omnibus (GEO) (https://www.ncbi.nlm.nih.gov/geo/query) through GEO Series accession number GSE109377 for RUNX1 Nalm6 and patients(3), and GSE117684 for the other data.

**Chromatin immunoprecipitation - PCR (ChIP-PCR)**

CHIP was performed according to the manufacturer’s instructions (SimpleChIP® Enzymatic Chromatin IP Kit (Agarose Beads), Cell signaling, #9002S). Approximately 4$\times$10^6^ of Nalm6 and REH cells were cross-linked in 1% formaldehyde. Cells were digested by micrococcal nuclease, then the nuclear pellet was suspended in chromatin immunoprecipitation (ChIP) buffer and sheared to a 150-900bp fragment size using the Covaris M200 sonicator with Duty Factor 5.0 and a total treatment time 16min (8 min followed by 1min incubation in ice and 8 min again). The sheared chromatin was incubated with antibodies against RUNX1 (ab23980), CBFA2T3 (ab234985), or normal rabbit IgG (Cell Signaling) **(Table S2)**. DNA was purified and qPCR was done using SYBRgreen reagent (Thermo Fisher Scientific). Primers used were as follows: human *RUNX1* promoter forward 5′- CCTGTGGTTTGCATTCAGTG-3′ and reverse 5′- ATTGAGATGGGCTGTGGAAA-3′ **(Table S1).** The binding capability was expressed as a percent of the total input chromatin. The positive control (H3, Cell signalling), provided within the kit, gave us an enrichment at 17.8% of the input in the Nalm6.

**Immunoblotting**

Cells were lysed for 30 min in 20 mM Tris-HCl, PH8.0, 150 mM NaCl, 0.5 mM EDTA and 1% Triton supplemented with protease inhibitors (04693113001, Roche). The samples were subjected to sodium dodecyl sulfate polyacrylamide gel electrophoresis (SDS-PAGE), transferred to nitrocellulose membranes, blocked in TBS/0.05% Tween-20 (TBST)/5% milk and probed with an appropriate antibody (**Table S2**). The membranes were washed and incubated 1 h at room temperature in TBST/5% milk containing the secondary antibody. The immunoblots were visualized with enhanced chemiluminescence Western blotting detection system (WBKLS0500, Merck Millipore) according to the manufacturer's instructions.

**Cell cycle**

REH cells were seeded at a concentration of 500,000 cells/mL. The cells were synchronized by two incubations with 40ng/mL Nocodazole (SML1665, Merck) for 16 hours. Between these two incubations, the cells were washed twice in PBS in order to release the cell cycle block. After the second incubation, the cells were washed twice in PBS to release the block and incubated back into cell culture medium. At 0h, 8h and 24h, the cells were then washed and fixed in 70% ethanol for 2 hours at 4°C. Subsequently, they were washed twice in PBS in order to remove all traces of ethanol. Finally, the cells were incubated 20 minutes in FxCycle^TM^-PI/RNase Staining Solution (Thermo Fisher – F10797) at room temperature. The fluorescence was measured through BD Accuri^TM^ C6 Plus Flow Cytometer, on channel FL-2. The percentage of each phase was calculated with FlowJo^TM^ V10 using Watson (Pragmatic) model.

**Apoptosis**

REH cells were washed twice to remove the culture medium. They were then incubated in 100µL Binding Buffer with 10µL of Annexin V-FITC (Annexin V-FITC Kit (Miltenyi Biotec)) for 15 minutes in the dark, at room temperature. Subsequently, the cells were washed twice with Binding Buffer and finally resuspended in 300µL Binding Buffer. Three microliters of Propidium Iodide solution were added to the sample prior to analysis through flow cytometer.

***Supplemental Table S1: List of primers used in this study.***

| Gene | Forward | Reverse |
| --- | --- | --- |
| ABL | CCAAGAAGGGGCTGTCCT | ATGCTACTGGCCGCTGAA |
| RUNX1 | ACAAACCCACCGCAAGTC | CATCTAGTTTCTGCCGATGTCTT |
| CBFA2T3 | TGAACTCGACATTGACGATCG | TCAGGAAGGGAATGACAAACG |
| KIT | CAAGTCAGTGCTGTCGGAAA | TGAATTCTTCCCCTTCCCTAA |
| RUNX1 promoter (ChIP-qPCR) | CCTGTGGTTTGCATTCAGTG | ATTGAGATGGGCTGTGGAAA |

***Supplemental Table S2: List of antibodies used.***

| Antigen | Reference | Uses |
| --- | --- | --- |
| CBFA2T3 | Ab33072 (abcam) | PLA, WB, Co-IP, ChIP-Seq |
| CBFA2T3 | Ab167326 (abcam) | PLA, WB |
| CBFA2T3 | Ab234985 (Abcam) | WB, Chip-qPCR |
| ETV6 | ab54705 (Abcam) | PLA |
| ETV6 | sc11382 (Santa Cruz biotechnology) | PLA |
| Halotag | G9281 (Promega) | Co-IP, WB |
| H3K4me1 | 07-436 (Merck Millipore) | ChIP-Seq |
| H3k4me3 | 04-745 (Merck Millipore) | ChIP-Seq |
| H3K27ac | ab4729 (Abcam) | ChIP-Seq |
| HSC70 | sc7298 (Santa Cruz biotechnology) | WB |
| Myc-tag | 2278P (cell signaling technology) | WB |
| Myc-tag | CST 71D10 (Cell Signaling Technology) | WB, PLA |
| RUNX1 | ab23980 (Abcam) | PLA, ChIP-Seq, ChIP-qPCR, Co-IP, WB |
| RUNX1 | Ab11035 (Abcam) | PLA |
| RUNX1 | MABD169 (EMD Millipore) | PLA |
| Flag | F1804 (Sigma) | WB |
| Flag | M8823, Sigma | Co-IP |
| KAT3A/CBP | ab2832 (Abcam) | PLA |
| KAT3B/EP300 | ab54984 (Abcam) | PLA |
| NCOR | ab24552 (Abcam) | PLA |
| HDAC1 | ab46985 (Abcam) | PLA |
| SIN3A | ab3479 (Abcam) | PLA |

**REFERENCES FOR THE SUPPLEMENTAL INFORMATION**

1. Fears S, Gavin M, Zhang DE, Hetherington C, Ben-David Y, Rowley JD, et al. Functional characterization of ETV6 and ETV6/CBFA2 in the regulation of the MCSFR proximal promoter. Proc Natl Acad Sci U S A. 4 mars 1997;94:1949‑54.

2. Kumar R, Cheney KM, McKirdy R, Neilsen PM, Schulz RB, Lee J, et al. CBFA2T3-ZNF652 corepressor complex regulates transcription of the E-box gene HEB. J Biol Chem. 4 juill 2008;283(27):19026‑38.

3. Debaize L, Jakobczyk H, Avner S, Gaudichon J, Rio A-G, Sérandour AA, et al. Interplay between transcription regulators RUNX1 and FUBP1 activates an enhancer of the oncogene c-KIT and amplifies cell proliferation. Nucleic Acids Res. 30 2018;46(21):11214‑28.

4. Debaize L, Jakobczyk H, Rio A-G, Gandemer V, Troadec M-B. Optimization of proximity ligation assay (PLA) for detection of protein interactions and fusion proteins in non-adherent cells: application to pre-B lymphocytes. Mol Cytogenet. 2017;10:27.

5. McLeay RC, Bailey TL. Motif Enrichment Analysis: a unified framework and an evaluation on ChIP data. BMC Bioinformatics. 1 avr 2010;11:165.

6. Nicol JW, Helt GA, Blanchard SG, Raja A, Loraine AE. The Integrated Genome Browser: free software for distribution and exploration of genome-scale datasets. Bioinformatics. 15 oct 2009;25(20):2730‑1.

7. Arnaud M-P, Vallée A, Robert G, Bonneau J, Leroy C, Varin-Blank N, et al. CD9, a key actor in the dissemination of lymphoblastic leukemia, modulating CXCR4-mediated migration via RAC1 signaling. Blood. 8 oct 2015;126(15):1802‑12.

8. Zhou X, Edmonson MN, Wilkinson MR, Patel A, Wu G, Liu Y, et al. Exploring genomic alteration in pediatric cancer using ProteinPaint. Nat Genet. janv 2016;48(1):4‑6.
