# Supplementary material for: Reduction of RUNX1 transcription factor activity by a CBFA2T3-mimicking peptide: application to B cell precursor acute lymphoblastic leukemia
Source: J Hematol Oncol. 2021 Mar 20;14:47. doi: 10.1186/s13045-021-01051-z (PMC7981807; doi:10.1186/s13045-021-01051-z)
Supplement: Supplementary file 2 — Additional file 2: Figures S1, S2 and S3 and their legends. [file 13045_2021_1051_MOESM2_ESM.pdf]

Figure S1

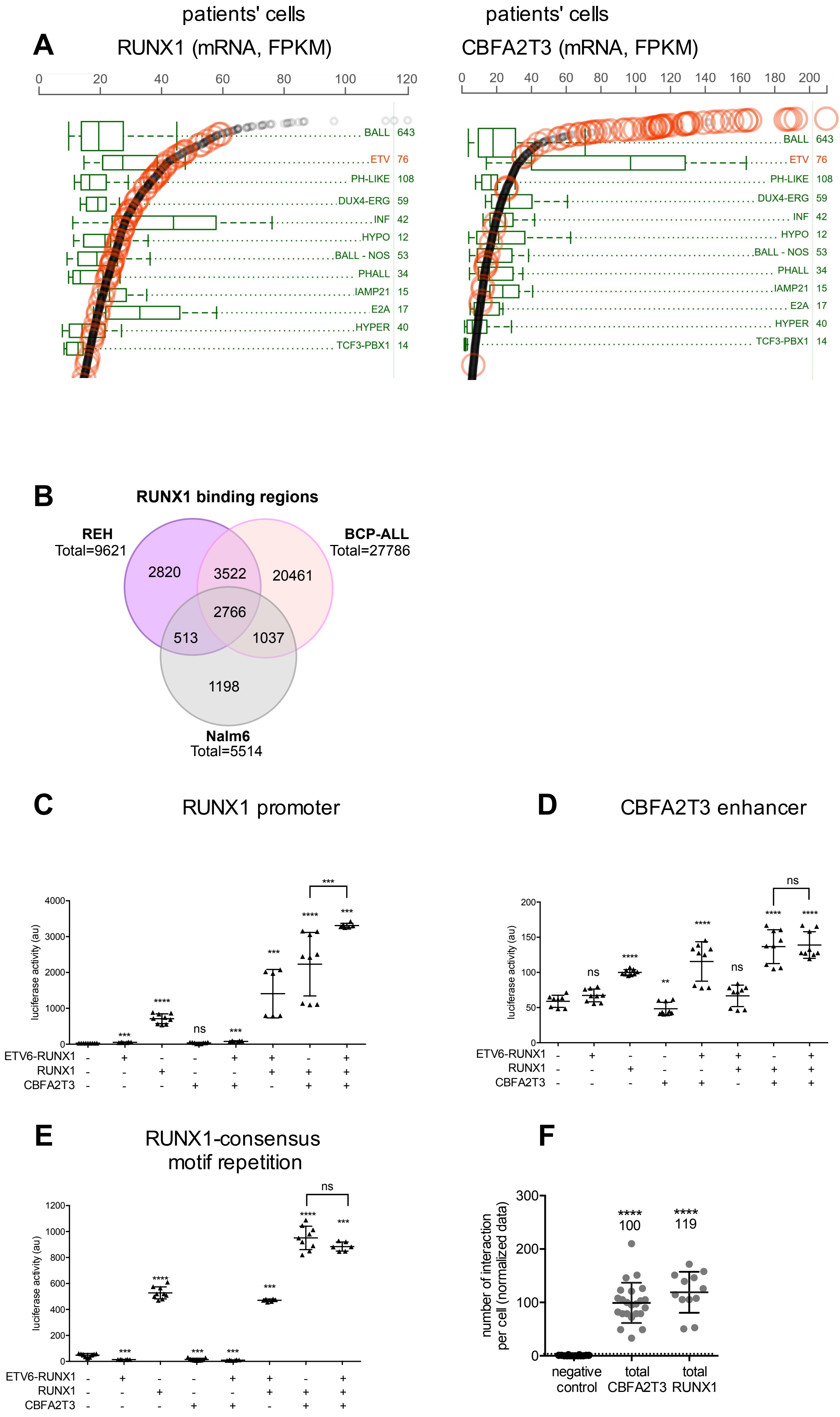

A
patients' cells
RUNX1 (mRNA, FPKM)
patients' cells
CBFA2T3 (mRNA, FPKM)
BALL 643
ETV 76
PH-LIKE 108
DUX4-ERG 59
INF 42
HYPO 12
BALL - NOS 53
PHALL 34
IAMP21 15
E2A 17
HYPER 40
TCF3-PBX1 14
B
RUNX1 binding regions
REH
Total=9621
BCP-ALL
Total=27786
Nalm6
Total=5514
2820
3522
20461
2766
513
1037
1198
C
RUNX1 promoter
D
CBFA2T3 enhancer
luciferase activity (au)
ETV6-RUNX1
RUNX1
CBFA2T3
E
RUNX1-consensus
motif repetition
F
number of interaction per cell (normalized data)
100
119
negative control
total CBFA2T3
total RUNX1

Figure S2

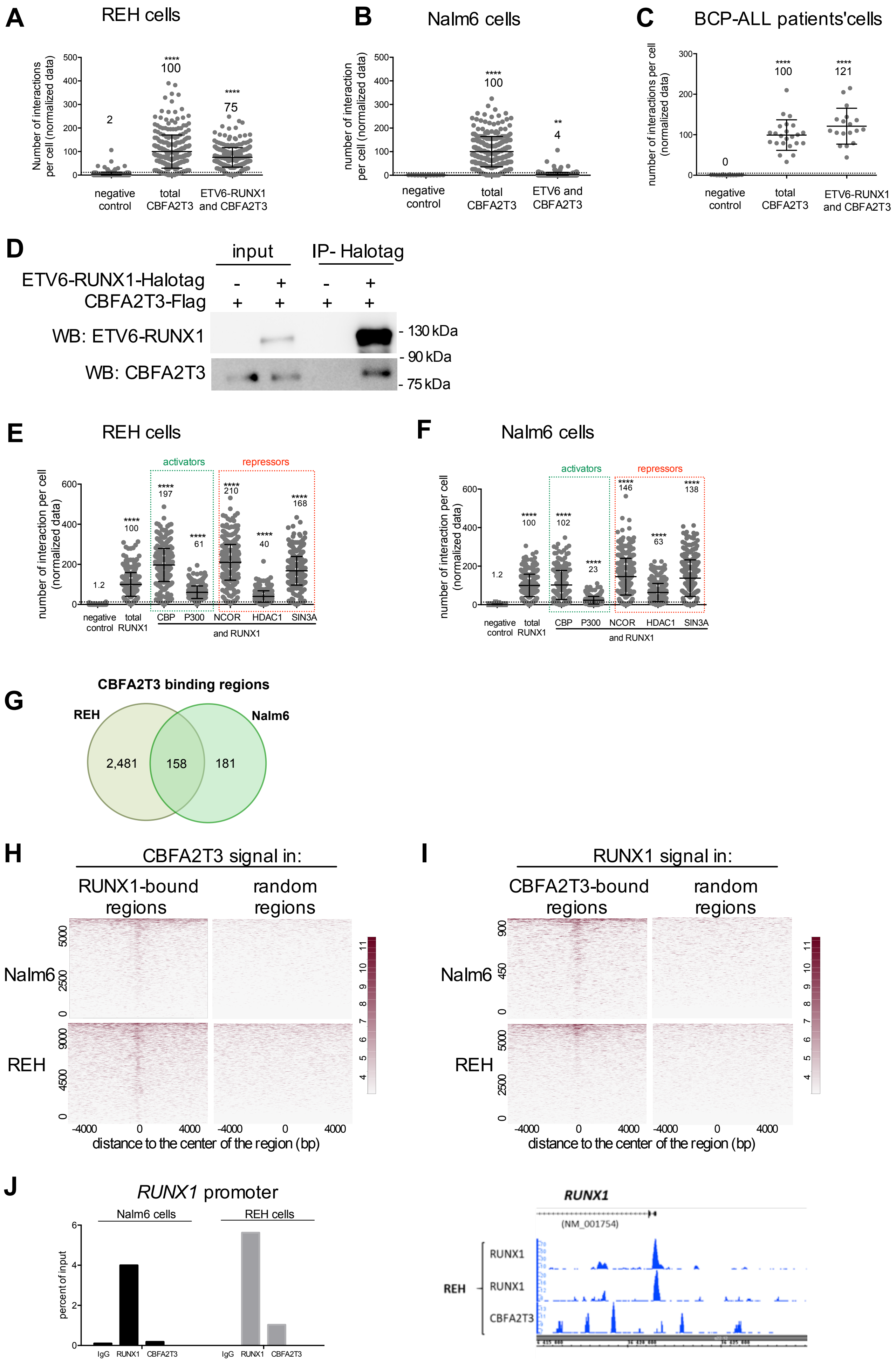
A
REH cells
Number of interactions per cell (normalized data)
negative control
total CBFA2T3
ETV6-RUNX1 and CBFA2T3
B
Nalm6 cells
number of interaction per cell (normalized data)
negative control
total CBFA2T3
ETV6 and CBFA2T3
C
BCP-ALL patients'cells
number of interactions per cell (normalized data)
negative control
total CBFA2T3
ETV6-RUNX1 and CBFA2T3
D
input
IP- Halotag
ETV6-RUNX1-Halotag
CBFA2T3-Flag
WB: ETV6-RUNX1
WB: CBFA2T3
130 kDa
90 kDa
75 kDa
E
REH cells
activators
repressors
number of interaction per cell (normalized data)
negative control
total RUNX1
CBP
P300
NCOR
HDAC1
SIN3A
and RUNX1
F
Nalm6 cells
activators
repressors
number of interaction per cell (normalized data)
negative control
total RUNX1
CBP
P300
NCOR
HDAC1
SIN3A
and RUNX1
G
CBFA2T3 binding regions
REH
Nalm6
2,481
158
181
H
CBFA2T3 signal in:
RUNX1-bound regions
random regions
Nalm6
REH
distance to the center of the region (bp)
I
RUNX1 signal in:
CBFA2T3-bound regions
random regions
Nalm6
REH
distance to the center of the region (bp)
J
RUNX1 promoter
Nalm6 cells
REH cells
percent of input
IgG
RUNX1
CBFA2T3
RUNX1
(NM_001754)
REH
RUNX1
RUNX1
CBFA2T3

Figure S3

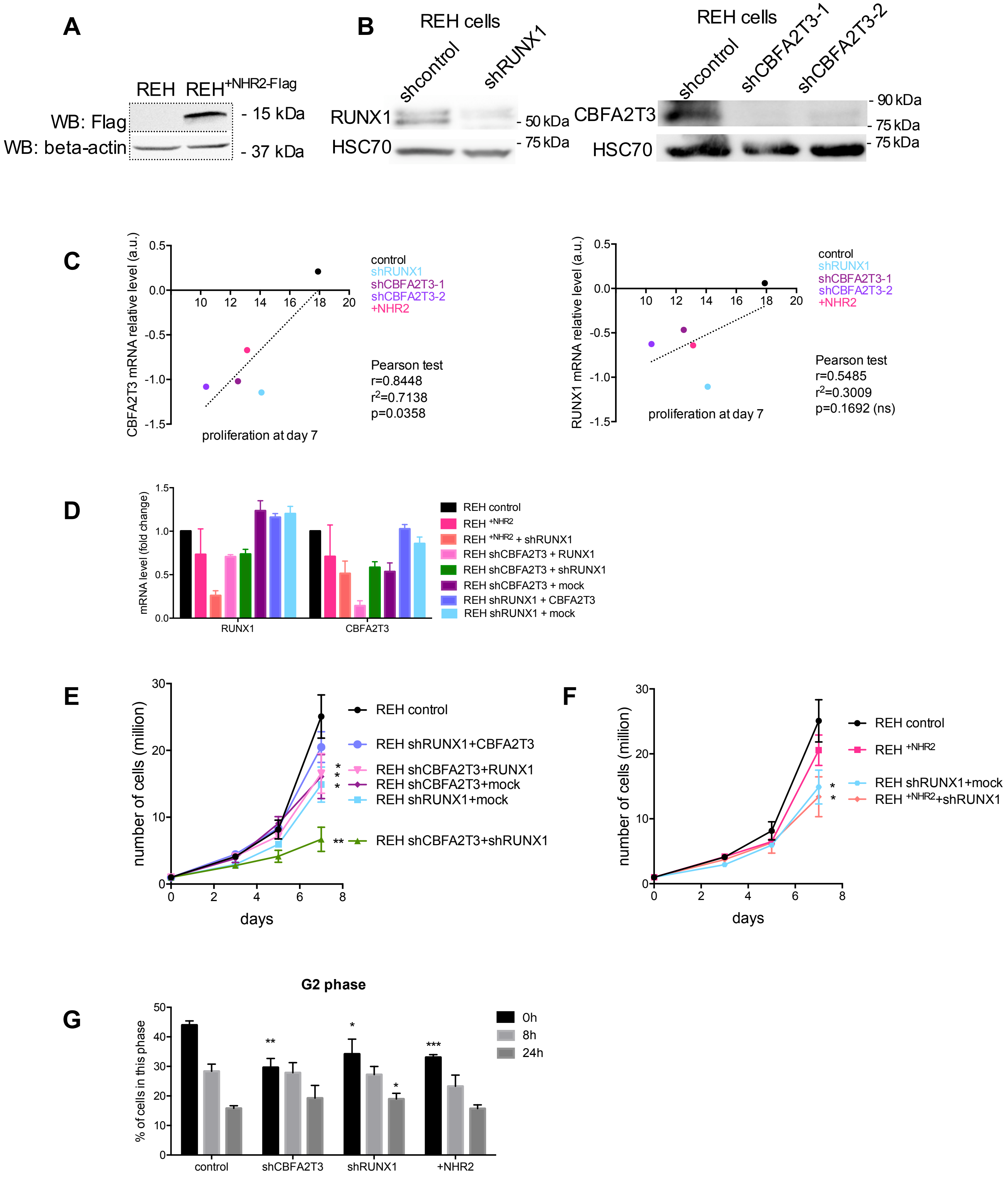
A
REH
REH+NHR2-Flag
WB: Flag
WB: beta-actin
- 15 kDa
- 37 kDa
B
REH cells
shcontrol
shRUNX1
RUNX1
HSC70
- 50 kDa
- 75 kDa
REH cells
shcontrol
shCBFA2T3-1
shCBFA2T3-2
CBFA2T3
HSC70
- 90 kDa
- 75 kDa
- 75 kDa
C
CBFA2T3 mRNA relative level (a.u.)
proliferation at day 7
control
shRUNX1
shCBFA2T3-1
shCBFA2T3-2
+NHR2
Pearson test
r=0.8448
r2=0.7138
p=0.0358
RUNX1 mRNA relative level (a.u.)
proliferation at day 7
control
shRUNX1
shCBFA2T3-1
shCBFA2T3-2
+NHR2
Pearson test
r=0.5485
r2=0.3009
p=0.1692 (ns)
D
mRNA level (fold change)
RUNX1
CBFA2T3
REH control
REH +NHR2
REH +NHR2 + shRUNX1
REH shCBFA2T3 + RUNX1
REH shCBFA2T3 + shRUNX1
REH shCBFA2T3 + mock
REH shRUNX1 + CBFA2T3
REH shRUNX1 + mock
E
number of cells (million)
days
REH control
REH shRUNX1+CBFA2T3
REH shCBFA2T3+RUNX1
REH shCBFA2T3+mock
REH shRUNX1+mock
REH shCBFA2T3+shRUNX1
F
number of cells (million)
days
REH control
REH +NHR2
REH shRUNX1+mock
REH +NHR2+shRUNX1
G
G2 phase
% of cells in this phase
control
shCBFA2T3
shRUNX1
+NHR2
0h
8h
24h

Supplemental Figure 1

Figure S1

A. Data of mRNA levels for *RUNX1* and *CBFA2T3* (expressed in Fragments Per Kilobase Million – FPKM) have been extracted from the St. Jude Children's Research Hospital RNA-Seq Pediatric Cancer Data Portal(8), and different subtypes of childhood BCP-ALL are shown. Data have been updated on June, 16th, 2020. BALL: B-cell Acute Lymphoblastic Leukemia, ETV: ETV6-RUNX1 BCP-ALL, PH-LIKE: Philadelphia chromosome–like BCP-ALL, INF: Infant BCP-ALL; HYPO: hypodiploid BCP-ALL, NOS: not otherwise specified, E2A: E2A-PBX1 BCP- ALL subtype, PHALL: BCR-ABL1 BCP-ALL, HYPER: Hyperdiploid BCP-ALL. Subtypes with less than 5 samples are not included.

B. Venn-diagrams presenting the intersection between RUNX1 ChIP-Seq binding regions in REH cells (common regions in both replicates), in Nalm6 cells (common regions in both replicates), and in bone marrow mononuclear cells isolated from 3 BCP-ALL patients. For the BCP-ALL patients, we considered only the common regions observed in 2 out of the 3 patients.

C-E. Luciferase assays with a plasmid containing the *RUNX1* promoter (C), *CBFA2T3* enhancer (D) and a repetition of RUNX1-consensus motif (E) upstream a minimal promoter and a luciferase ORF, in presence of ETV6-RUNX1, RUNX1 and CBFA2T3 expressing plasmids in HEK293T cells. Luciferase levels (Firefly luciferase/Renilla luciferase) are represented using a scatter dot plot indicating the means and S.D. ns: non-significant, ** p<0.01, *** p<0.001, **** p<0.0001 in Mann-Whitney tests compared to the control situation (first left condition).

F. Quantitation of endogenous total RUNX1 and total CBFA2T3 proteins in BCP-ALL patient cells measured by PLA presented with the mean values ± S.D. A negative control is also included. The data are normalized against total CBFA2T3. The mean value is indicated above each plot. The positive threshold value is represented by the dotted line as described in (Debaize et al, molecular cytogenetics 2017). **** p<0.0001 in Fisher's exact test compared to the negative control condition.

Supplemental Figure 2

Figure S2.

A-C: Quantitation of ETV6-RUNX1, ETV6 and CBFA2T3 protein co-localization per nucleus–visualized by PLA dots in REH cells (A), Nalm6 cells (B), and BCP-ALL patient cells (C) presented with the mean values ± S.D. Positive controls (total CBFA2T3) and negative controls were included. The data are normalized against total CBFA2T3. The mean value is indicated above each plot. The positive threshold value is represented by the dotted line as described in (Debaize et al, molecular cytogenetics 2017). ** p<0.01, **** p<0.0001 in Fisher's exact test compared to the negative control condition.

D: Co-immunoprecipitation (IP) using anti-Halotag antibody in HEK293T cells expressing ETV6-RUNX1-Halotag and CBFA2T3-Flag plasmids. Western blots were performed with RUNX1 and CBFA2T3 antibodies.

E-F: Quantitation of RUNX1 and various activators or repressor protein co-localization per nucleus–visualized by PLA dots in REH cells (A), and Nalm6 cells (B) presented with the mean values ± S.D. Positive controls (total RUNX1) and negative controls were included. The data are normalized against total RUNX1. The mean value is indicated above each plot. The positive threshold value is represented by the dotted line as described in (Debaize et al, molecular cytogenetics 2017).

G. Venn-diagrams presenting the intersection between CBFA2T3 ChiP-Seq binding regions in REH cells and in Nalm6 cells.

H,I. (H) heatmap of Chip-Seq CBFA2T3 signals into Chip-Seq RUNX1-bound regions or random regions in Nalm6 cells (top line) and in REH cells (bottom line). (I) heatmap of Chip-Seq RUNX1 signals into Chip-Seq CBFA2T3-bound regions or random regions in Nalm6 cells (top line) and in REH cells (bottom line).

J. left panel: Chromatin Immunoprecipitation-qPCR with IgG, RUNX1, CBFA2T3 and Histone H3 (positive control) antibodies in Nalm6 and REH cells on the *RUNX1* promoter. The results are expressed as percentage of the input. Right panel: ChIP-Seq profiles across the human *RUNX1* gene. Genomic tracks display ChIP-Seq profiles for RUNX1 and CBFA2T3 from REH cells. ChIP-Seq reads were aligned to the reference human genome version GRCh37 (hg19). The figure is centered on the *RUNX1* promoter.

Supplemental Figure 3

Figure S3.

A, B. Western blots of total protein extracts from REH control cells and REH expressing CBFA2T3$^{NHR2\text{-}Flag}$ cells (REH^{+NHR2}) (A), REH shRUNX1 (B), REH shCBFA2T3-1 (B), REH shCBFA2T3-2 (B). Western blots were performed with Flag, RUNX1 and CBFA2T3 antibodies, and beta-actin or HSC70 for the loading control.

C. XY plots showing the relative mRNA level of *CBFA2T3* and *RUNX1* according to the proliferation rate expressed as the number of cells (expressed in million) at day 7 from the experiment of Figure 7 A and B. Each dot corresponds to the mean on the mRNA level and of the proliferation rate for each condition. A Pearson correlation test has been performed.

D. Quantification of *CBFA2T3* and *RUNX1* mRNA level in the different cell lines of (E) and (F).

E, F. Proliferation curves for REH^{shcontrol}, REH$^{shRUNX1+mock}$, REH$^{shCBFA2T3+mock}$, REH$^{shCBFA2T3+shRUNX1}$, REH$^{shCBFA2T3+RUNX1}$, REH$^{shRUNX1+CBFA2T3}$ (E), and for REH$^{shRUNX1+mock}$, REH^{+NHR2} cells, REH $^{shRUNX1+NHR2}$ cells (F). Three experiments are represented for each condition. For more readibility, statistical analyses have been run only for the last day (day 7) and compared to the condition REH^{shcontrol}. * p<0.05; ** p<0.005.

G. Progression in G2 phase after 0, 8 and 24hours after nocodazole-block release in REH^{shcontrol}, REH^{shRUNX1} REH^{shCBFA2T3} REH^{+NHR2} cells**.** The results are expressed as the percentage of cells in G2-phase. * $p<0.05$; ** $p<0.005$; *** $p<0.0005$.
